# Sacred Moments During Mentorship

**DOI:** 10.1007/s11606-025-09399-5

**Published:** 2025-02-03

**Authors:** Sanjay Saint

**Affiliations:** 1 Patient Safety Enhancement Program, University of Michigan and Veterans Affairs (VA) Ann Arbor Healthcare System, Ann Arbor, MI USA; 2 Department of Internal Medicine, University of Michigan Medical School, Ann Arbor, MI USA; 3 VA Ann Arbor Healthcare System, Ann Arbor, MI USA; 4 University of Michigan, Ann Arbor, MI 48109 USA

I was taking the news poorly. I was a junior faculty member at a new institution hoping to receive a federal career development award to launch my research career. I had 3 chances. The first submission was well-received by the review panel, and the suggestions addressable. I responded with what I thought was a strong resubmission. My resulting score, however, was far worse than the first attempt. The program officer discouraged a final submission. I was devastated and embarrassed, assuming my research career was over. Until one conversation altered my perspective.

My mentor came to the door of my office one morning and said he had heard the gloomy news and asked how I was doing. I was honest with him: not well. He said the following while standing at the door to my office (paraphrasing as this happened nearly 25 years ago):

*“Sanjay, you should know something. I have spoken with your other mentors and our division chief. We all think highly of you and will support you regardless of the outcome of this or that grant. The work you propose in patient safety is important. Review panels can be fickle. And their opinions are not an accurate reflection of who you are. They are often wrong. Anyone who writes grants for a living has experienced what you have. What counts is shots on goal. Just take another shot.”*

My relief was immediate. I was not aware that I was still supported by my mentors or colleagues. I was unaware that my experience was common. I also appreciated the re-framing my mentor provided by going from outcome to effort. I went from being despondent to determined. I prepared for my next shot! Fast forward several months and my next shot on goal went in and my research career was launched. Things have gone well in the intervening 25 years. I have used that same saying with several other junior faculty who have faced similar circumstances. Without the care and consideration of my mentor that day, however, I do not know how long it would have taken me to gain the confidence to try again.

What I experienced with my mentor was a sacred moment: a brief period when time stood still, I felt deeply seen and understood by him, and, at least to me, was unforgettable.^1^ I remember the setting and what he said. And what followed was a sense of peace. Dr. Suzanne Koven recently wrote “a mentor is someone who has more imagination about you than you have about yourself”^2^ — what an apt description!

Sacred moments—spoken of in a psychological sense, rather than theological—is a term coined by Ken Pargament as he described interactions between clinical psychologists and patients.^3^ Since then, sacred moments have shown to be common in the hospital setting as well.^4^ These moments have been referred to as “sudden intimacies” that can occur with strangers at times of crisis, and that connect people in meaningful ways.^5^ In interviewing clinicians about these moments, the number that said these interactions are “the reason they went into medicine” is astounding.^4^

As I have learned more about this concept and reflect on my academic career, I realized sacred moments occur in mentoring relationships as well, as I learned first-hand. When I asked several of my colleagues to share such experiences with me, this is what they wrote.

A surgical houseofficer:

*One month into my Fulbright Fellowship in Italy, I was anxious. Moving to a new country, unsuccessfully attempting to contact a remote medical team, and working with a local mentor I had yet to meet in person was taking a toll. Further, the stakes were high having taken a non-traditional approach to my protected academic time. During our first Zoom check-in with my U.S. mentor, I dove into my project to-dos. After several minutes he gently interrupted, asking: “How is the transition going? How are you doing?” He gave me space to be honest about my concerns, laying the groundwork for addressing the challenges. While many mentor-mentee relationships focus solely on professional goals, the more successful ones build mutual respect and appreciation for the individuals involved—similar to how we establish positive relationships with patients. This foundation set the stage for our professional rapport and academic work together.*

A project manager:

*My first job in research was at Johns Hopkins as a research assistant, and I was intimidated by the accomplished physician researchers with whom I worked. The only physician I had ever met growing up was my primary care doctor, and now I was helping manage multi-million dollar projects. I had no idea what I was doing, but I dove in. After a few years the physician I worked for (who happens to now be a medical school dean!) asked if I wanted to first author a paper on our work. I was confused – this wasn’t usually asked of a research assistant. What she said next has stuck with me to this day over a decade later: “We think of you as a co-investigator. That’s how much you bring to the team.” I was stunned. That was not how I saw myself. It changed my self-perception. It’s funny how a single sentiment can shift your view of yourself and what you are capable of doing.*

Finally, a hospitalist, describing a sacred moment he had as a mentor:

*Despite my early career stage, I agreed to mentor a medical student. Occasionally the student alluded to feeling anxious about their progress. In one meeting, something seemed especially off. I asked how life was outside of the hospital. The student paused for a moment then shared, in a torrent of words, that they were internally grappling with their sexuality, that their home communities were not accepting of this, that it was impacting all aspects of life and taking up immense space. We sat together in silence. No spoken words could express how grateful I was that they shared this deeply personal information with me, but I attempted to convey it anyway. They thanked me for being open and avoiding judgment. Shifting from professional growth to focus on human connection proved to be the sacred moment in that mentoring relationship.*

None of the above had shared such moments with a professional colleague until I asked them about it. These are often moments that have redefined one’s career, yet colleagues often do not mention them. Perhaps by first cultivating such moments and then discussing them we can make them more common.

I end with a quote from J Randall Curtis, MD (a palliative care leader who died of amyotrophic lateral sclerosis on February 6, 2023) who I had the honor of working with during my fellowship:

*“Before my diagnosis, I used to think of my legacy as the papers I had published and the impact that my research has had on the field of medicine. Since my diagnosis, my thinking has changed. I now see my legacy as the people I have mentored and helped mentor and the people that they have mentored. This vision of legacy gives me much more joy and happiness than my old vision of legacy.”*

There is indeed something sacred in mentoring others.
